# Anti-Obesity Effect of Pine Needle Extract on High-Fat Diet-Induced Obese Mice

**DOI:** 10.3390/plants10050837

**Published:** 2021-04-21

**Authors:** Eun A. Kim, Ju-Hwan Yang, Eun-Hye Byeon, Woong Kim, Dawon Kang, Jaehee Han, Seong-Geun Hong, Deok-Ryong Kim, Sang-Je Park, Jae-Won Huh, Hyeonsook Cheong, Seung-Pil Yun, Dong-Kun Lee

**Affiliations:** 1 Department of Physiology and Convergence Medical Science, Institute of Health Sciences, Gyeongsang National University Medical School, Jinju 52727, Korea; ggomangi00@naver.com (E.-H.B.); dawon@gnu.ac.kr (D.K.); jheehan@gnu.ac.kr (J.H.); hong149@gnu.ac.kr (S.-G.H.); 2 Department of Integrative Biological Sciences & BK21 FOUR Educational Research Group for Age-Associated Disorder Control Technology, Chosun University, Gwangju 61452, Korea; gadak2@naver.com; 3 Department of Biochemistry and Convergence Medical Science, Institute of Health Sciences, Gyeongsang National University Medical School, Jinju 52727, Korea; drkim@gnu.ac.kr; 4 National Primate Research Center, Korea Research Institute of Bioscience and Biotechnology, Cheongju 28116, Korea; parksj@kribb.re.kr (S.-J.P.); huhjw@kribb.re.kr (J.-W.H.); 5 Department of Functional Genomics, KRIBB School of Bioscience, Korea University of Science & Technology (UST), Daejeon 34113, Korea; 6 Department of Biomedical Science, College of Natural Sciences, Chosun University, Gwangju 61452, Korea; hscheong@chosun.ac.kr; 7 Department of Pharmacology and Convergence Medical Science, Institute of Health Sciences, Gyeongsang National University Medical School, Jinju 52727, Korea

**Keywords:** hypothalamus, pine needle extract, melanocortin system, obesity, energy balance, proopiomelanocortin, brown adipose tissue, white adipose tissue, fat tissue metabolism

## Abstract

Background: Obesity due to an excessive intake of nutrient disturbs the hypothalamus-mediated energy metabolism subsequently develops metabolic disorders. In this study, we investigated the effect of pine needle extract (PNE) on the hypothalamic proopiomelanocortin (POMC) neurons involved in the regulation of energy balance via melanocortin system and fat tissue metabolism. Methods: We performed electrophysiological and immunohistochemical analyses to determine the effect of PNE on POMC neurons. Mice were fed a normal or high-fat diet for 12 weeks, then received PNE for the last 2 weeks to measure the following physiological indices: Body weight, food intake, fat/lean mass, glucose metabolism, and plasma leptin levels. In addition, changes of thermogenic, lipolytic, and lipogenetic markers were evaluated in brown adipose tissue (BAT) and white adipose tissue (WAT) by western blotting, respectively. Results: PNE increased hypothalamic POMC neuronal activity, and the effect was abolished by blockade of melanocortin 3/4 receptors (MC3/4Rs). PNE decreased body weight, fat mass, plasma leptin levels, and improved glucose metabolism after high-fat-induced obesity. However, PNE did not change the expression of thermogenic markers of the BAT in HFD fed groups, but decreased only the lipogenetic markers of WAT. This study suggests that PNE has a potent anti-obesity effect, inhibiting lipogenesis in WAT, even though HFD-induced leptin resistance-mediated disruption of POMC neuronal activity.

## 1. Introduction

Endeavors to overcome obesity and type 2 diabetes have become a global issue, given the billions of people suffering from metabolic and related diseases [1,2]. In terms of energy homeostasis, development of obesity is due to an imbalance between energy intake and consumption [3]. The hypothalamus is a key regulator of energy balance between energy intake and expenditure by integrating circulating hormonal and nutritional signals [4,5,6,7]. Among the hypothalamic nuclei, the arcuate nucleus (ARC) plays a major role to control energy balance [4,6,8]. In the ARC, two types of primary order neurons—including anorexigenic proopiomelanocortin (POMC) and orexigenic neuropeptide Y (NPY)/agouti-related peptide (AgRP) neurons—reciprocally regulate energy balance by sensing glucose and adiposity signals [5,9,10]. In particular, POMC neurons downregulate feeding behaviors and increase energy expenditures, while NPY/AgRP neurons play opposite roles [8,9,10].

In the melanocortin system, the POMC neurons project to melanocortin receptor subtype 4 (MC4R)-expressing neurons in the paraventricular nucleus (PVN) and modulate the feeding behavior and energy expenditure by releasing α-MSH [11,12,13,14,15]. In addition, POMC neurons are strongly linked to interscapular brown adipose tissue (BAT) thermogenesis and beigeing of the white adipose tissue (WAT) which are closely related to energy expenditure [16,17,18,19]. However, melanocortin system dysfunction can lead to obesity and its associated diseases such as type 2 diabetes mellitus and cardiovascular disorders, even neurodegenerative disorders [3,4,5,6,7,20,21].

The red pine (*Pinus densiflora* Sieb et. Zucc.) is a plant that is well known for its nutritional and pharmaceutical qualities. As a medicinal plant, pine needles contain abundant beneficial compounds, including proteins, vitamins, minerals, and many other chemicals [22]. There is some evidence that pine needle extract may be helpful to prevent the onset of metabolic disorders and related conditions such as cardiovascular disease, cancer, diabetes, and hypertension [23]. Among the ingredients of the pine needle extracts, polyphenolic antioxidants are known to be effective against diseases such as diabetes and fatty liver disease [23,24]. Terpenoids—other pine needle components—downregulate blood cholesterol and glucose levels [23,25]. However, the role of pine needle extract (PNE) on the regulation of energy balance via the hypothalamus remains unclear. In this study, we applied pine needle powder obtained by juicing pine needles and then freeze-dried it to investigate whether this crude compound affected hypothalamic POMC neurons involved in the regulation of energy balance.

## 2. Results

### 2.1. PNE Increases C-Fos Expression in Both ARC and PVN of the Hypothalamus

**Figure 1:**
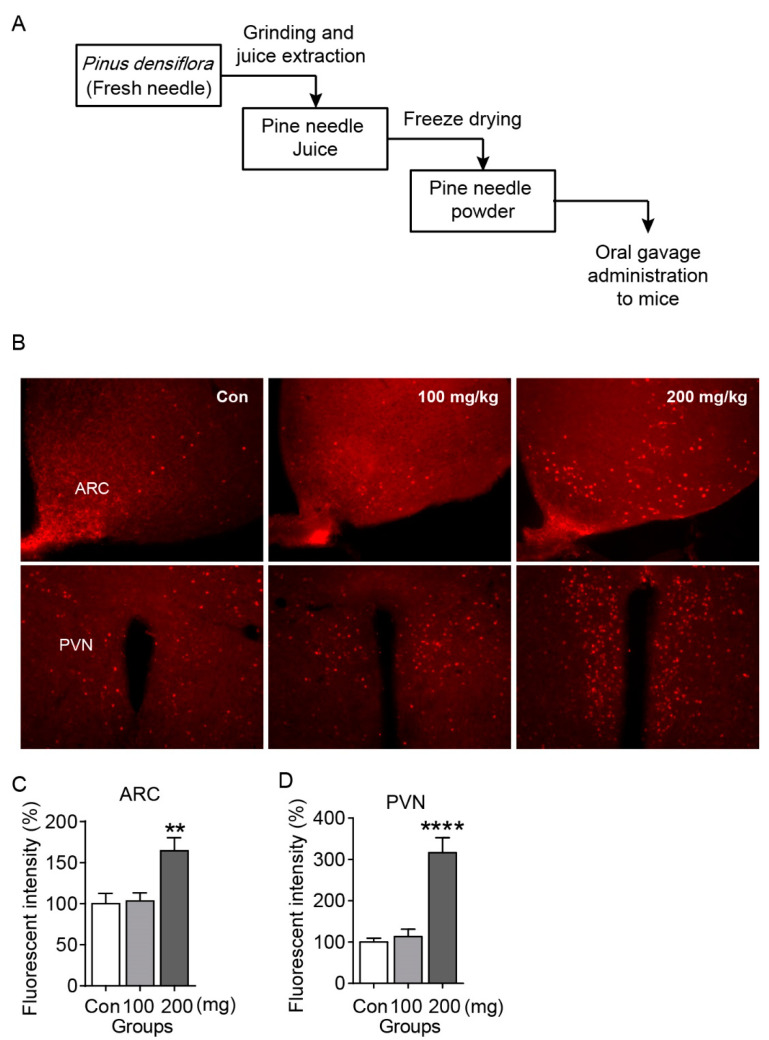
Oral gavage injection of PNE increased c-fos expression in both the ARC and PVN. (**A**) Schematic illustration of the PNE extract method. (**B**) Immunohistochemical staining of the c-fos expression in the ARC and PVN after 200 mg/kg of PNE treatment. (**C**,**D**) Quantitative analyses of fluorescent intensity of c-fos expression in the ARC and PVN after PNE treatments (Con, *n* = 6; PNE 100 mg/kg, *n* = 8; PNE 200 mg/kg, *n* = 9), respectively. ** *p* < 0.01, **** *p* < 0.0001 vs. Con. All data are shown as mean ± SEM.

Fresh pine needles were collected from *Pinus densiflora* to make juice. The obtained juice was freeze-dried with pine needle powder. The PNE obtained through this process was mixed with water and administered orally to mice (Figure 1A). To determine the role of PNE related in the hypothalamic regulation of energy balance, we measured c-fos expression levels in the ARC and PVN by immunostaining of the mouse brain slices. The c-fos expression was significantly increased by single oral gavage administration of PNE (200 mg/kg) in both ARC and PVN after 1 h (Figure 1B–D), but not in the ventromedial hypothalamus (data not shown). These data suggest that acute oral injection of PNE activates the melanocortin system in the hypothalamus.

### 2.2. Blockade of PNE-Evoked Depolarization of ARC POMC Neurons by MC4Rs Inhibition

**Figure 2:**
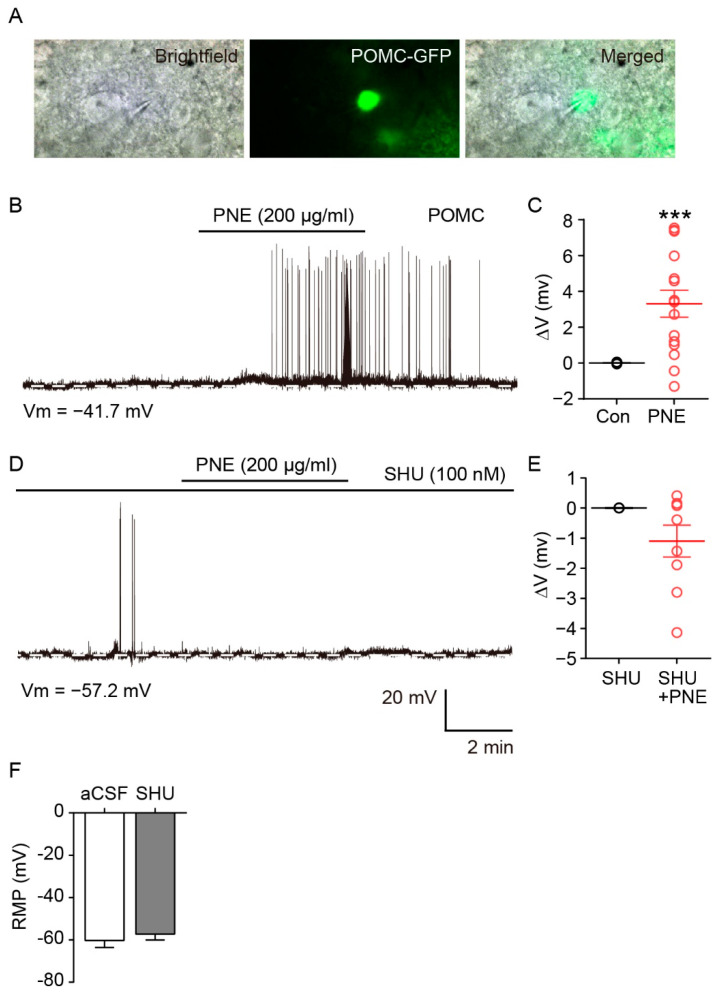
PNE depolarized ARC POMC neurons, but this elevation was inhibited by the MC4R blockade. (**A**) Brightfield, fluorescent (eGFP), and a merged image of targeted POMC neuron in the ARC for the patch-clamp recording. (**B**) Representative recording trace showing the PNE effect on POMC neurons. (**C**) Quantitative analysis of the PNE effect on POMC neurons. Among the 15 POMC neurons, 10 neurons were depolarized after PNE treatment. (**D**) Representative recording trace showing the effect of blockade of MC4R after PNE treatment on POMC neurons. (**E**) Quantitative analysis of blockade of MC4R on POMC neurons after PNE treatment. In all of 9 recorded POMC neurons, PNE-evoked depolarization was blocked by SHU-9119 (100 nM). (**F**) Single effect of SHU-9119 did not change the membrane potential at 100 nM concentration. *** *p* < 0.001 vs. Con. All data are shown as mean ± SEM. SHU, SHU 9119.

**Figure 3:**
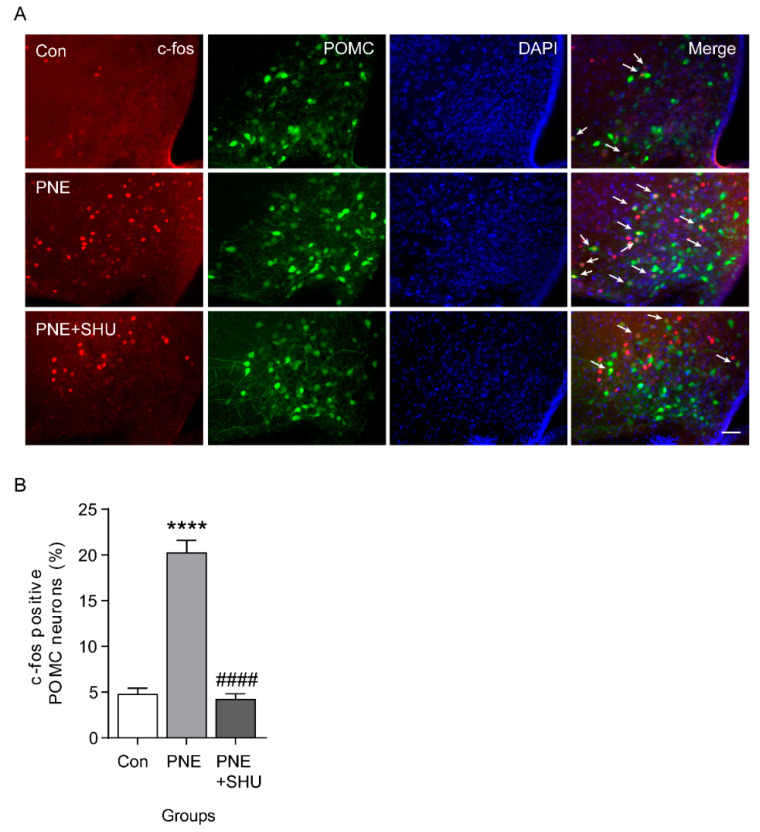
Blockade of MC3/4Rs inhibited PNE-induced c-fos expression in the ARC POMC neurons. (**A**) Images of fluorescence microscopy showing that POMC neurons (green) expressed c-fos (red) after oral gavage administration of PNE (200 mg/kg, middle panel). However, PNE-mediated elevation of c-fos expression in the POMC neurons was inhibited by MC4R antagonist SHU-9119. SHU-9119 was given intraperitoneally before 30 min to oral gavage administration of PNE. Scale bar: 50 μm. (**B**) Quantitative analysis of POMC and c-fos colocalization after single or combination treatment of PNE and PNE+SHU9119 treatment (Con, *n* = 6; PNE, *n* = 5; PNE+SHU-9119, *n* = 7). **** *p* < 0.0001 vs. Con; ^####^ *p* < 0.0001 vs. PNE. All data are shown as mean ± SEM.

Since PNE activates both ARC and PVN in the hypothalamus, we tested whether PNE activates anorexigenic POMC neurons, which are major neuronal type of the melanocortin system that regulates energy balance. For cell-specific visualization of POMC neurons, POMC-Cre: POMC-eGFP was used in this study (Figure 2A). The electrophysiological results showed that PNE (200 μg/mL) significantly depolarized the ARC POMC neurons (10 of 15 neurons, Figure 2B,C), while this elevation was completely blocked by MC3/4Rs antagonist SHU-9119 (100 nM, 9 neurons, Figure 2D,E). However, the POMC neuronal activity did not change by SHU-9119 treated alone (0.1 mg/kg, i.p., Figure 2F). Similarly, immunohistochemical data showed that blockade of MC3/4Rs by SHU-9119 significantly decreased PNE-mediated upregulation of c-fos expression in ARC POMC neurons (Figure 3A,B). These data suggest that PNE plays a critical role in regulating the melanocortin system via MC3/4Rs, which is commonly expressed in ARC POMC and PVN neurons.

### 2.3. PNE Does Not Change the ARC NPY/AgRP Neuronal Activity in the Hypothalamus

**Figure 4:**
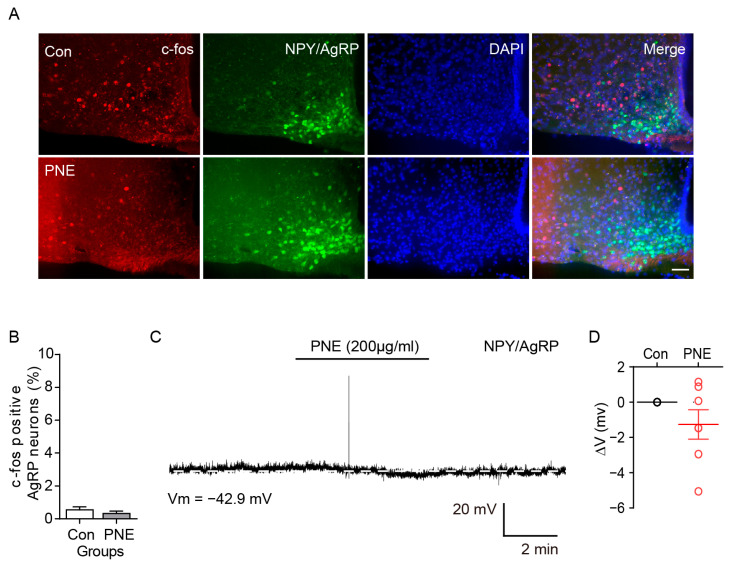
PNE did not change the NPY/AgRP neuronal activity in the hypothalamus. (**A**) Images of fluorescence microscopy showing that NPY/AgRP neurons (green) expressed c-fos (red) after oral gavage administration of PNE (200 mg/kg, upper and middle panels). Scale bar: 50 μm. (**B**) Quantitative analysis of NPY/AgRP neurons and c-fos colocalization after PNE treatment (Con, *n* = 3; PNE, *n* = 7). (**C**) Representative recording trace showing the PNE effect on NPY/AgRP neurons. (**D**) Quantitative analysis of the PNE effect on NPY/AgRP neurons (*n* = 6 neurons). All data are shown as mean ± SEM.

Since PNE activates POMC neurons, we tested whether PNE changes the activity of orexigenic NPY/AgRP neurons in the hypothalamic melanocortin system. For cell-specific visualization of NPY/AgRP neurons, AgRP-Cre: CRISPR/Cas9-EGFP was used (Figure 4A). The immunohistochemical data showed that NPY/AgRP neurons rarely expressed c-fos after PNE (200 mg/kg) treatment (Figure 4A,B). In addition, electrophysiological results showed that PNE (200 μg/mL) did not change membrane potential of the ARC NPY/AgRP neurons (Figure 4C,D). These data suggest that PNE affect POMC neurons, but not NPY/AgRP neurons in the melanocortin system; therefore, this may contribute to the anorexigenic role by activating the ARC POMC neurons.

### 2.4. PNE Reduces Fat Mass but Not Feeding Behavior in HFD Fed Mice

**Figure 5:**
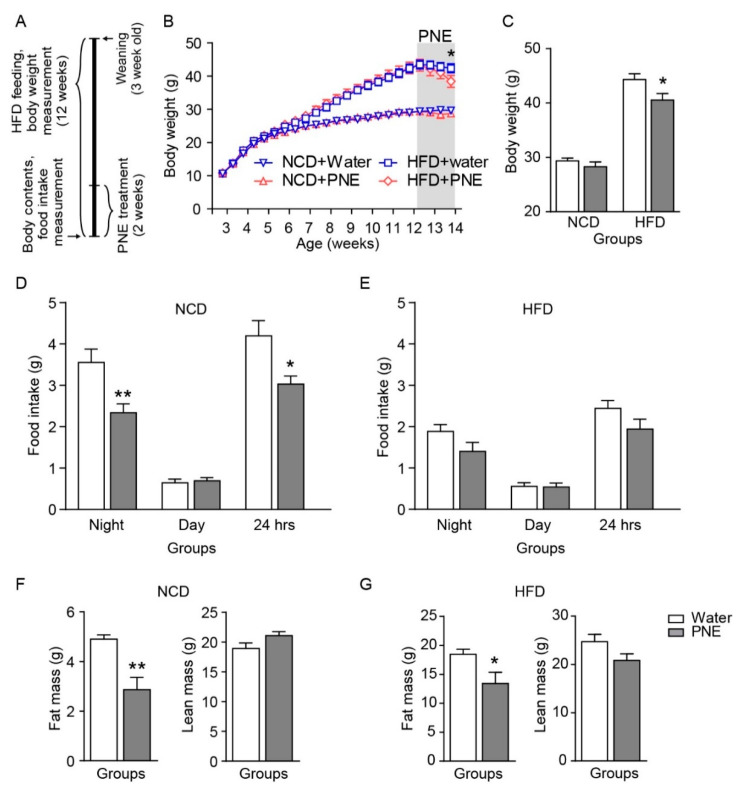
PNE effect on food intake and body weight after normal chow and high-fat diet. (**A**) Schematic timeline depicting food intake and bodyweight measurement after HFD was fed. (**B**) Except for NCD, HFD-fed mice exhibited reduced body weight by daily oral gavage administration of PNE (NCD+water, *n* = 14; NCD+PNE, *n* = 13; HFD+water, *n* = 16; HFD+PNE, *n* = 15, respectively) for 2 weeks. (**C**) Body weight, (**D**) food intake for normal chow diet (NCD+water, *n* = 16; NCD+PNE, *n* = 11), (**E**) food intake for high-fat diet (HFD+water, *n* = 18; HFD+PNE, *n* = 15), (**F**) fat and lean mass (NCD+water, *n* = 4; NCD+PNE, *n* = 3) for NCD fed groups, and (**G**) fat and lean mass (*n* = 6 per group, respectively) for HFD fed groups. * *p* < 0.05, ** *p* < 0.01 vs. water. All data are shown as mean ± SEM.

Previous studies showed that POMC neurons have an anorexigenic role and enhance energy expenditure [26]. To identify the anti-obesity effect of PNE, each group of mice was provided either a 60% kcal high-fat diet (HFD) or 10% kcal normal chow diet (NCD) for 12 weeks, respectively. Body weights were measured twice weekly after weaning. Ten weeks after HFD feeding, mice were given PNE (200 mg/kg, once per day) by oral gavage administration for 2 weeks (Figure 5A). The amount of food intake was measured during the night (from 7 p.m. to 7 a.m.) and day (from 7 a.m. to 7 p.m.) on the last day of the experiment, respectively. Food intake and fat mass, but not body weight, significantly changed in the NCD group (Figure 5B–D,F). However, PNE significantly decreased body weight and body fat mass, but did not reduce food intake in the HFD fed groups (Figure 5B,C,E,G). These data suggest that PNE decreased food intake via melanocortinergic POMC neurons in normal conditions, but that PNE may assist body weight loss by reducing body fat mass, without affecting feeding behaviors in the obese state.

### 2.5. PNE Improves Glucose Tolerance by Regulating Leptin Levels in HFD-Induced Obese Mice

**Figure 6:**
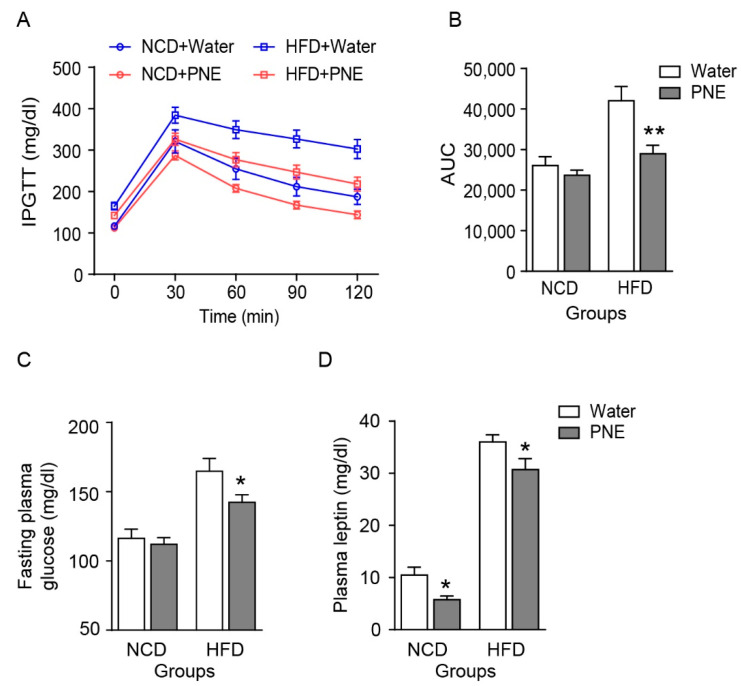
PNE improved glucose and leptin levels after HFD feeding. (**A**) Intraperitoneal glucose tolerance test (IPGTT, 2 g/kg) for plasma glucose kinetics after PNE oral injection (NCD+water, *n* = 8; NCD+PNE, *n* = 10; HFD+water, *n* = 14; HFD+PNE, *n* = 17, respectively). The mice fasted overnight prior to the IPGTT. (**B**) The area under the curve for IPGTT. (**C**) Changes in fasting plasma glucose levels in NCD- and HFD-fed mice. (**D**) Changes in plasma leptin levels 1 h after PNE treatment. * *p* < 0.05, ** *p* < 0.01 vs. water. All data are shown as mean ± SEM.

Hypothalamic POMC neurons sense blood glucose and leptin levels involved in glucose and fat tissue metabolism [26,27,28]. Previous studies showed that disrupted glucose sensing in POMC neurons caused the development of type 2 diabetes in mice with HFD-induced obesity [26,27,28,29]. Therefore, we performed an intraperitoneal glucose tolerance test (IPGTT) after overnight fasting in obese mice, which were provided an HFD for 12 weeks. The IPGTT results showed that the administration of PNE improved glucose tolerance and fasting glucose levels in obese mice (Figure 6A–C). In both NCD-fed and HFD-fed groups, leptin levels were decreased by PNE administration (Figure 6D). Previous studies have demonstrated that PNE treatment reduced total cholesterol, triglyceride, and leptin levels by suppressing differentiation of adipocytes and reduced adipose tissue mass [30,31]. Therefore, these data suggest that PNE affects plasma leptin levels via POMC neurons in normal conditions; however, PNE may involve fat tissue metabolism to regulate leptin levels under HFD-induced leptin resistance.

### 2.6. Body Condition-Dependent Regulation of Adipose Tissue Metabolism by PNE

**Figure 7:**
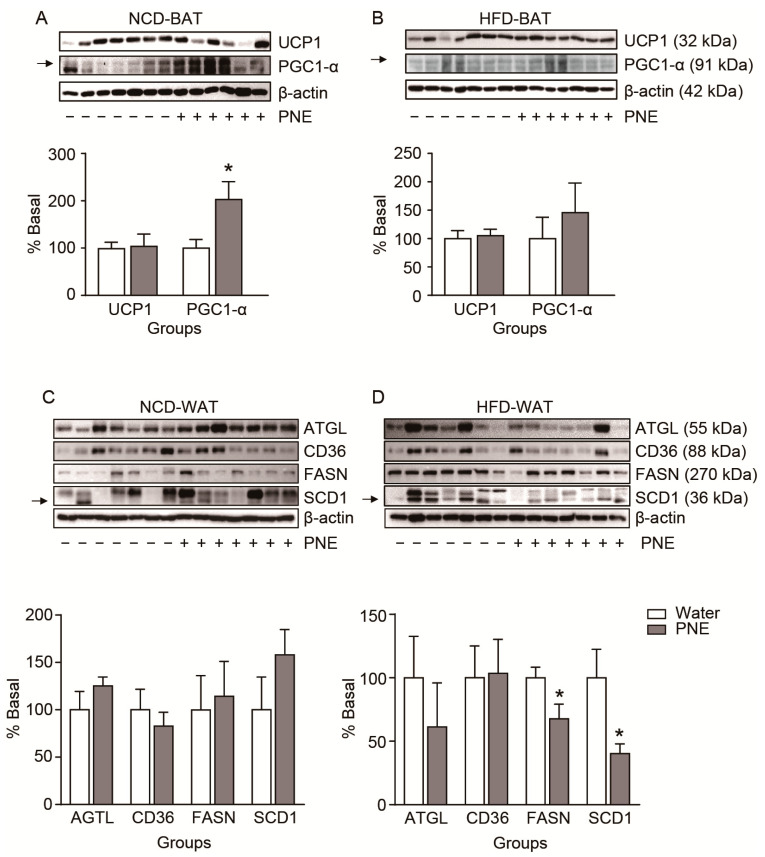
Changes of fat tissue metabolism after PNE treatment in both NCD and HFD groups. Expression levels of BAT thermogenesis markers after PNE treatment in (**A**) NCD (NCD+water, *n* = 7; NCD+PNE, *n* = 6) and (**B**) HFD (HFD+water, *n =* 7; HFD+PNE, *n =* 6) groups. Expression levels of WAT lipolysis (ATGL, CD36) and lipogenesis (FASN, SCD1) markers after PNE treatment in (**C**) NCD (NCD+water, *n =* 7; NCD+PNE, *n =* 7) and (**D**) HFD (HFD+water, *n =* 7; HFD+PNE, *n* = 7) groups. PNE upregulates BAT thermogenesis in NCD group, but downregulates WAT lipogenesis in HFD group, respectively. * *p* < 0.05 vs. water. All data are shown as mean ± SEM.

**Figure A1:**
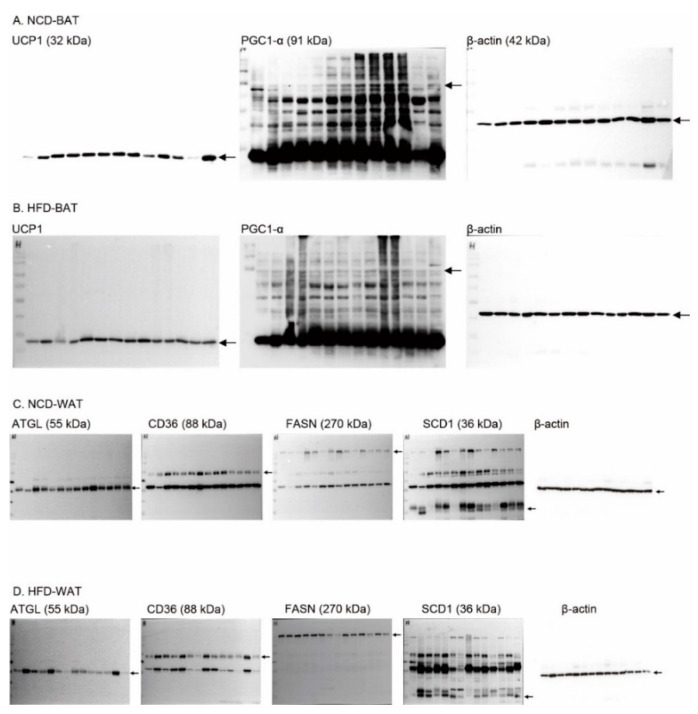
The full-length western blot images for thermogenic, lipogenic, and lipolytic markers. (**A**) UCP1, PGC1-α, and β-actin expression levels in the BAT after NCD fed. (**B**) UCP1, PGC1-α, and β-actin expression levels in the BAT after HFD fed. (**C**) ATGL, CD36, FASN, and SCD1 expression levels in the WAT after NCD fed. (**D**) ATGL, CD36, FASN, and SCD1 expression levels in the WAT after HFD feeding.

POMC neurons have multi-synaptic connections that regulate lipid metabolism [29,32,33]. Therefore, we investigated whether PNE could affect to fat tissue metabolism, including BAT thermogenesis, WAT lipolysis, and lipogenesis. Two types of functional markers were used for each group to evaluate BAT thermogenesis (uncoupling protein 1, UCP1; peroxisome proliferator-activated receptor-γ coactivator 1-α, PGC1-α), WAT lipolysis (adipose triglyceride lipase, AGTL; cluster of differentiation 36, CD36), and WAT lipogenesis (fatty acid synthase, FASN; stearoyl-CoA desaturase 1, SCD1) after PNE administration, respectively. As shown in Figure 7, PNE administration increased PGC1-α expression of BAT in NCD (Figure 7A), but not in the HFD-fed group (Figure 7B). Further, we investigated whether PNE was involved in the white fat tissue metabolism. After PNE administration, only the expression levels of lipogenetic markers (FASN and SCD1) were significantly decreased in the HFD-fed group (Figure 7D), but did not change in the NCD group (Figure 7C). In addition, lipolytic markers did not change in either groups. These data suggest that PNE is also involved in fat tissue metabolism by itself, even under the disturbed leptin signaling condition by HFD-induced obesity. The full-length western blot images in Figure 7 are shown in Figure A1 in Appendix A.

## 3. Discussion

The complex interaction of energy metabolism and appetite is modulated by the hypothalamus and is essential for sustaining life in living organisms. Dysfunction of ARC neurons in the hypothalamus that are involved in regulation of energy balance can lead to obesity and its associated metabolic disorders [1,3,4,5,6,7,10]. In this study, we investigated the physiological role of PNE in regulating the energy balance via hypothalamic POMC neurons. We found that oral administration of PNE activated hypothalamic nuclei, both the ARC and PVN, but not the VMH. In the melanocortin system, two types of ARC neurons—the POMC and NPY/AgRP—project to MC4R-expressing PVN neurons and regulate PVN neuronal activity by releasing α-MSH and γ-aminobutyric acid (GABA), respectively [32,34,35]. Moreover, several studies have shown that modulation of POMC neuronal activity is closely related to the MC3 and MC4Rs, although POMC neurons innervate MC4R-expressing neurons in the PVN [36,37]. Hence, we expected that PNE would affect the melanocortin system via ARC POMC neuronal activity since both ARC and PVN neurons are activated by PNE administration. As shown in Figure 1, oral gavage administration of PNE increased c-fos expression levels in the ARC and PVN. In addition, the electrophysiological and immunohistochemical results in Figure 2 and Figure 3 have provided evidence that PNE administration activates the ARC POMC neurons, and that the elevation was blocked by a blockade of POMC neuronal activity by the MC3/4Rs antagonist. These findings suggest that POMC neurons respond to PNE and may assist regulation of energy balance via interplay with PVN. However, NPY/AgRP neurons did not respond to the PNE (Figure 4). These data suggest that PNE plays a critical role in POMC neurons, but not NPY/AgRP neurons, and may downregulate food intake or promote energy expenditure.

As shown in Figure 5, administration of PNE affected food intake in normal conditions, but not in a high-fat-induced obese state, even with body weight loss. In particular, PNE reduced the fat mass in the high-fat-induced obese group. These results demonstrated that PNE has a critical role in regulating the energy balance via the melanocortin pathway that initiated from POMC in normal condition, but this pathway hindered by HFD-induced obesity. In addition, PNE improved glucose homeostasis and leptin levels in the HFD-induced obese state (Figure 6). Therefore, we expected that PNE would affect fat tissue metabolism. Previous studies found that the melanocortin system affects various physiological functions of fat tissue [33,38]. In addition, PNE has a pivotal role in decreasing differentiation of adipocyte cells [31]. In particular, BAT thermogenesis is closely linked to energy expenditures via sympathetic output which primarily originates within hypothalamic nuclei [38,39]. The functional significance of the melanocortin pathway in the regulation of BAT metabolism was previously reported in Pomc KO and Mc4r-KO mice [38,40]. Among BAT thermogenesis markers, PNE administration increased PGC 1-α levels in mice fed an NCD, but did not change in mice fed an HFD (Figure 7A,B). However, PNE decreased expression levels of lipogenesis markers, but did not change the expression levels of lipolysis markers in WAT in mice fed an HFD (Figure 7D). These data suggest that PNE is positively involved in the regulation of BAT metabolism through ARC POMC neurons in normal conditions, but only affected to lipogenesis in HFD-induced obesity. In brief, PNE activates the melanocortin pathway to enhance BAT thermogenesis, consequently reducing the fat mass in normal conditions, but PNE appears to be only involved in the lipogenesis of the WAT in HFD-mediated leptin resistance.

PNE affects energy homeostasis through its active ingredients. Various species of pine needle have abundant polyphenols, including proanthocyanidins, quercetin, (+)-catechin, ferulic acid, ρ-coumaric acid, vanillic acid, and caffeic acid [41]. The beneficial effects on obesity by polyphenols are well established [41,42]. Dietary polyphenols also have therapeutic potential against energy-rich diet-induced hypothalamic dysfunction and metabolic disease by reducing the inflammatory response and oxidative stress in the hypothalamus [43]. Furthermore, among the active ingredients contained within pine needles, proanthocyanidins enhance hypothalamic leptin/STAT3 signaling and Pomc gene expression, as well as reducing hyperphagia and improving leptin resistance in obese rats [44]. Though the melanocortin system modulates fat tissue metabolism, PNE also may affect fat tissue physiology by itself. In fact, previous studies showed that HFD mediated enlargement of white adipocytes and enhancement of leptin release in adipose tissue, consequently causing leptin resistance [45,46,47]. Leptin resistance is an important index to the interruption of the ARC POMC neuronal function, which is mediated by the leptin receptor signaling pathway [5,7,13,28]. As shown Figure 7, while PNE affects lipogenesis in the leptin resistance model, it seems to not affect the BAT thermogenesis via the melanocortin system. This is crucial evidence suggesting that PNE requires the leptin/STAT3 signaling pathway in POMC neurons to participate in BAT thermogenesis [38,39,40,44]. In addition, PNE hindered lipogenesis by downregulating expression of lipogenetic markers under the leptin resistance condition (Figure 7). The role of PNE on obesity remains largely unknown, but our study showed that PNE is involved in both regulation of fat tissue metabolism and melanocortin system. However, it seemed that PNE only affects to the lipogenesis under leptin resistance.

**Figure 8:**
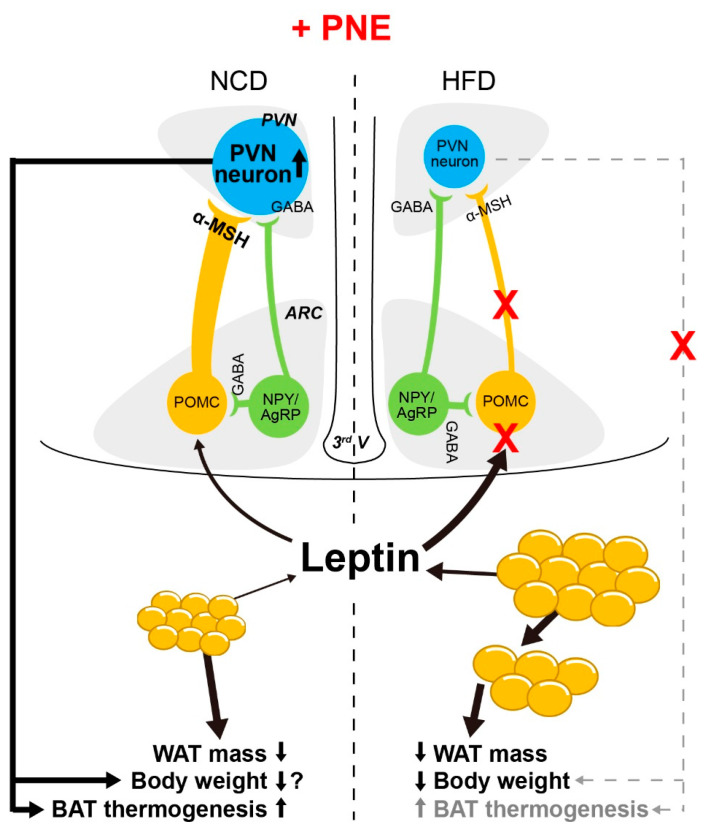
Putative mechanism of PNE on melanocortin system and fat tissue metabolism. Oral gavage administration of PNE enhances ARC POMC neuronal activity and subsequently activates PVN MC4R neurons. This series activation of the melanocortin system may contribute to the enhancement of energy expenditures and reduction of fat mass and food intake in normal conditions. However, PNE hinders lipogenesis of WAT and reduces body weight even though PNE cannot affect to the melanocortin system under the HFD-induced leptin resistance condition.

Taken together, these results demonstrate that PNE elevates energy expenditures and reduces food intake through activation of POMC neurons in the hypothalamus (Figure 8). In addition, PNE could be involved in fat tissue metabolism, independently of the melanocortin system in an HFD-induced obese condition. Even though PNE is a crude extract, the anti-obesity effects of PNE may emerge as a useful therapeutic for use in individuals suffering from obesity-related metabolic diseases.

## 4. Materials and Methods

### 4.1. Animals

All experimental and animal care protocols were approved by the Gyeongsang National University Institution Animal Care and Use Committee (GNU IACUC, GNU-170504-M0019) and performed in accordance with the National Institutes of Health (NIH) guidelines and with a scientifically reviewed protocol (GLA-100917-M0093). Mice used in these experiments included POMC-Cre (stock # 005965, Jackson Laboratory, Bar Harbor, ME, USA) and POMC-eGFP (stock # 009593, Jackson Laboratory, Bar Harbor, ME, USA), AgRP-IRES-Cre (stock # 012899, Jackson Laboratory, Bar Harbor, ME, USA), CRISPR/Cas9 (stock # 026175, Jackson Laboratory, Bar Harbor, ME, USA), which are mixed C57BL/6, FVB, and 129 strain backgrounds. CRISPR/Cas9 mice were used for the Cre recombinase-dependent expression of eGFP.

### 4.2. Preparation of Pine Needle Extract

Cultivated fresh pine needles (*Pinus densiflora*) were collected during spring (from February to April) from Goksung, Jeonlanam-Do, South Korea. The collected pine needles were cleaned several times using distilled water and the moisture on the surface was completely removed. Then pine needles were juiced using a specially self-made juicer (Inwha Precision and Samkwang, Gwangju, Korea). After juicing, PNE were freeze-dried with a vacuum freeze dryer (Samwon SFDSF12, Busan, Korea) at −70 to −85 °C. The dried PNE was pulverized using an ultrafine particle mill (Korea patent R&D institution, Busan, Korea) to make powder. The collected PNE powder was dissolved in distilled water and administered orally to mice. The PNE used in this study was donated by Dr. Cheong at Chosun National University.

### 4.3. Measurement of Body Weight, Food Intake, and Body Mass Composition

Mice were fed either an NCD or an HFD containing 10% or 60% kcal (Research Diets, Inc., New Brunswick, NJ, USA), respectively. The body weight of the mice was measured twice weekly while either NCD or HFD was provided. Periods of food intake were measured from 7 p.m. to 7 a.m. and from 7 a.m. to 7 p.m. on the last day of the experiment, respectively. Body mass composition was measured once at the end of the experiments using the EchoMRI analyzer (EchoMRI LCC., Houston, TX, USA). All mice were randomly assigned to either the NCD or HFD group.

### 4.4. Slice Preparation and Electrophysiological Recordings

Transverse sectioned brain slices (200 μm thickness) were prepared by vibratome (7000smz-2; Campden Instruments, Loughborough, UK). The pipette solution contained 130 mM of K-gluconate, 5 mM of CaCl_2_, 10 mM of EGTA, 10 mM of HEPES, 2 mM of MgATP, 0.5 mM of Na_2_GTP, and 10 mM of phosphocreatine. To record membrane potentials, brain slices were placed in a recording chamber and superfused with artificial cerebrospinal fluid (containing: 113 mM of NaCl, 3 mM of KCl, 1 mM of NaH_2_PO_4_, 26 mM of NaHCO_3_, 2.5 mM of CaCl_2_, 1 mM of MgCl_2_, and 5 mM of glucose in 95% O_2_/5% CO_2_) at 1.5–2 mL/min. The recording chambers were placed on the stage of an upright and infrared differential interference contrast microscope (Olympus BX51WI; Olympus, San Jose, CA, USA), which was mounted on a Gibraltar X-Y table. The prepared brain slices were visualized by infrared microscopy with a 40X water immersion objective. Whole-cell current-clamp recordings were obtained from visualized ARC POMC neurons of the POMC-eGFP mice brain slices at a holding potential of −70 mV. Electrophysiological signals were low-pass filtered at 2–5 kHz, saved on a desktop PC and analyzed offline with pClamp 11 software (Molecular Devices, San Jose, CA, USA). For each recording, membrane potentials measured every 30 s were considered as single data points. We compared a total of 10 data points before and after the application of PNE were compared using paired *t*-tests. Membrane potentials were recorded using a multi-clamp 700 B (Molecular Devices, San Jose, CA, USA) in the whole-cell configuration. All recordings were conducted at 30 ± 2 °C.

### 4.5. Immunofluorescence Staining

Male 5–6-week-old mice were used to prepare the brain slices. The mice were anesthetized with avertin and transcardially perfused with a pre-perfusion solution (10 U/mL of heparin in PBS). Isolated mice brains were incubated in 4% paraformaldehyde (in phosphate-buffered saline (PBS)) overnight at 4 °C. The next day, the brain samples were sectioned with a vibratome (Leica Microsystems, Buffalo Grove, IL, USA) in 40-μm slices and stored at 4 °C in PBS. For immunofluorescence staining, the slices were washed three times in 0.5% TritonX/PBS for 10 min. The slices were blocked with 1 mL of 0.5% TritonX/BSA for 1 h and then washed three times with PBS for 10 min. The slices were labeled with an anti-c-fos rabbit antibody (1:1000, Cell Signaling, Danvers, MA, USA) with 2% BSA/PBS overnight. After three washes with PBS, Alexa fluor 594 anti-rabbit secondary antibody (1:1000, Abcam, Danvers, MA, USA) with 2% BSA/PBS was labeled for 2 h at RT. All images were acquired using an Olympus fluorescent microscope (Olympus, Tokyo, Japan).

### 4.6. IPGTT

After 16 h of fasting, glucose solution (2 mg/kg, i.p.) was given to the mice. We then measured blood glucose levels using a glucose meter (MEDISENSOR, Deagu, Korea) at 0, 30, 60, 90, and 120 min. A blood sample was taken from the mouse’s tail vein, and the first drop of blood was discarded. The area under the curve (AUC) from the IPGTT was calculated using the trapezoid rule.

### 4.7. Blood Plasma Leptin Assay

At 10 a.m., blood samples from (non-fasting) mice were collected into ethylene glycol tetraacetic acid (EGTA)-coated tubes (Becton, Dickinson and Company, Franklin Lakes, NJ, USA) to measure plasma leptin levels. Collected blood samples were centrifuged at 3000 rpm for 10 min to isolate the blood plasma. Plasma leptin levels were quantified using the ELISA assay kits purchased from Crystal Chem (Crystal Chem, Elk Grove Village, IL, USA).

### 4.8. Western Blot

Isolated interscapular BAT samples were homogenized in a lysis buffer containing 10 mM of Tris-HCl, pH 7.4, 5 mM of NaF, 1 mM of Na_3_VO_4_, 1 mM of EDTA, and 1 mM of EGTA on ice for 1 h and then sonicated. The total lysates were centrifuged at 13,000 rpm for 30 min at 4 °C to separate the supernatant protein for western blot analysis [48] and the pellet, which contains nuclei and large debris, was discarded. The concentration of the solubilized proteins in the supernatant fraction was determined using a BCA Protein Assay (Pierce, Rockford, IL, USA). The protein samples in the supernatant (20 μg) were separated on 10% SDS-PAGE cells and transferred to a methanol-activated polyvinylidene difluoride (PVDF) membrane (BioRad, Hercules, CA, USA). The membrane was blocked with a blocking buffer containing 5% skim milk in a mixture of Tris-buffered saline and 0.1% Tween-20 (TBST) and washed three times for 10 min. After washing, the membrane was probed with rabbit primary antiserums against UCP1 (1:1000, Abcam), CD36 (1:1000, Abcam), ATGL (1:1000, Cell Signaling), FASN (1:1000, Cell Signaling), and SCD1 (1:1000, Cell Signaling) for 18 h at 4 °C with mild shaking, respectively. The membrane was washed again and incubated with a peroxidase-labeled goat anti-rabbit IgG (1:3000; Thermo Fisher Scientific, Tewksbury, MA, USA) under the same conditions as the primary antiserum for 1 h at RT. Next, immunoreactive protein bands were detected using iBright western blot imaging systems (Thermo Fisher Scientific) with an enhanced chemiluminescence (ECL) solution (Pierce). The same membrane was stripped and probed with β-actin (1:1000, Santa Cruz Biotechnology, Dallas, TX, USA) to normalize the blots. Immunoreactive protein bands were semi-quantified using a digital imaging camera and the NIH Image 1.62 software, as previously described [49].

### 4.9. Statistics

Statistical analyses were performed using an unpaired *t*-test or one-way ANOVA with Tukey multiple comparison test (GraphPad Prism 4.03). Data were considered significantly different when the *p*-value was <0.05. All statistical results are given as mean ± SEM.

## Data Availability

Not applicable.

## References

[1] Romieu I., Dossus L., Barquera S., Blottière H.M., Franks P.W., Gunter M., Hwalla N., Hursting S.D., Leitzmann M., Margetts B. (2017). Energy balance and obesity: What are the main drivers?. Cancer Causes Control.

[2] Lumeng C.N., Saltiel A.R. (2011). Inflammatory links between obesity and metabolic disease. J. Clin. Investig..

[3] James O., Holly R., John C. (2012). Energy Balance and Obesity. Circulation.

[4] Dietrich M.O., Horvath T.L. (2013). Hypothalamic control of energy balance: Insights into the role of synaptic plasticity. Trends Neurosci..

[5] Waterson M.J., Horvath T.L. (2015). Neuronal Regulation of Energy Homeostasis: Beyond the Hypothalamus and Feeding. Cell Metab..

[6] Harrold J.A. (2004). Hypothalamic control of energy balance. Curr. Drug Targets.

[7] Timper K., Brüning J.C. (2017). Hypothalamic circuits regulating appetite and energy homeostasis: Pathways to obesity. Dis. Model Mech..

[8] Chen Y., Lin Y.C., Kuo T.W., Knight Z.A. (2015). Sensory detection of food rapidly modulates arcuate feeding circuits. Cell.

[9] Belgardt B.F., Okamura T., Brüning J.C. (2009). Hormone and glucose signalling in POMC and AgRP neurons. J. Physiol..

[10] Jobst E.E., Enriori P.J., Cowley M.A. (2004). The electrophysiology of feeding circuits. Trends Endocrinol. Metab..

[11] Kim G.W., Lin J.E., Valentino M.A., Colon-Gonzalez F., Waldman S.A. (2011). Regulation of Appetite to Treat Obesity. Expert Rev. Clin. Pharmacol..

[12] Fenselau H., Campbell J.N., Verstegen A.M., Madara J.C., Xu J., Shah B.P., Resch J.M., Yang Z., Mandelblat-Cerf Y., Livneh Y. (2017). A rapidly acting glutamatergic ARC → PVH satiety circuit postsynaptically regulated by α-MSH. Nat. Neurosci..

[13] Baldini G., Phelan K.D. (2019). The melanocortin pathway and control of appetite–progress and therapeutic implications. J. Endocrinol..

[14] Millington G.W. (2007). The role of proopiomelanocortin (POMC) neurones in feeding behaviour. Nutr. Metab. (Lond.).

[15] Pritchard L.E., Turnbull A.V., White A. (2002). Pro–opiomelanocortin processing in the hypothalamus: Impact on melanocortin signaling and obesity. J. Endocrinol..

[16] Caron A., Lee S., Elmquist J.K., Gautron L. (2018). Leptin and brain-adipose crosstalks. Nat. Rev. Neurosci..

[17] Zhang W., Bi S. (2015). Hypothalamic Regulation of Brown Adipose Tissue Thermogenesis and Energy Homeostasis. Front. Endocrinol. (Lausanne).

[18] Zhang Y., Rodrigues E., Li G., Gao Y., King M., Carter C.S., Tumer N., Cheng K.Y., Scarpace P.J. (2011). Simultaneous POMC gene transfer to hypothalamus and brainstem increases physical activity, lipolysis and reduces adult-onset obesity. Eur. J. Neurosci..

[19] Rodrigues K.C.D.C., Pereira R.M., de Campos T.D.P., de Moura R.F., da Silva A.S.R., Cintra D.E., Ropelle E.R., Pauli J.R., de Araújo M.B., de Moura L.P. (2018). The Role of Physical Exercise to Improve the Browning of White Adipose Tissue via POMC Neurons. Front. Cell. Neurosci..

[20] Mejido D.C.P., Peny J.A., Vieira M.N.N., Ferreira S.T., De Felice F.G. (2020). Insulin and leptin as potential cognitive enhancers in metabolic disorders and Alzheimer’s disease. Neuropharmacology.

[21] Picone P., Di Carlo M., Nuzzo D. (2020). Obesity and Alzheimer disease: Molecular bases. Eur. J. Neurosci..

[22] Seo H., Lee N.H., Ryu S. (2014). Antioxidant and antiapoptotic effects of pine needle powder ingestion and endurance training in high cholesterol-fed rats. J. Exerc. Nutr. Biochem..

[23] Park G.Y., Paudyal D., Hwang I.D., Tripathi G., Yang Y.K., Cheong H.S. (2008). Production of fermented needle extracts from red pine and their Functional characterization. Biotechnol. Bioprocess. Eng..

[24] Li H., Wang Z., Xu Y., Sun G. (2016). Pine polyphenols from Pinus koraiensis prevent injuries induced by gamma radiation in mice. PeerJ.

[25] Proshkina E., Plyusnin S., Babak T., Lashmanova E., Maganova F., Koval L., Platonova E., Shaposhnikov M., Moskalev A. (2020). Terpenoids as Potential Geroprotectors. Antioxidants.

[26] Toda C., Santoro A., Kim J.D., Diano S. (2017). POMC Neurons: From Birth to Death. Annu. Rev. Physiol..

[27] Dodd G.T., Michael N.J., Lee-Young R.S., Mangiafico S.P., Pryor J.T., Munder A.C., Simonds S.E., Bringing J.C., Zhang Z.Y., Cowley M.A. (2018). Insulin regulates POMC neuronal plasticity to control glucose metabolism. Elife.

[28] Lee D.K., Jeong J.H., Chun S.K., Chua S.J., Jo Y.H. (2015). Interplay between glucose and leptin signaling determines the strength of GABAergic synapses at POMC neurons. Nat. Commun..

[29] Parton L.E., Ye C.P., Coppari R., Enriori P.J., Choi B., Zhang C.Y., Xu C., Vianna C.R., Balthasar N., Lee C.E. (2007). Glucose sensing by POMC neurons regulates glucose homeostasis and is impaired in obesity. Nature.

[30] Park G.Y., Paudyal D., Park Y.M., Lee C.S., Hwang I.D., Tripathi G.R., Cheong H.S. (2008). Effects of Pine Needle Extracts on Plasma Cholesterol, Fibrinolysis and Gastrointestinal Motility. Biotechnol. Bioprocess. Eng..

[31] Jeon J.R., Kim J.Y. (2006). Effects of pine needle extract on differentiation of 3T3-L1 preadipocytes and obesity in high-fat diet fed rats. Biol. Pharm. Bull..

[32] Sohn J.W., Elmquist J.K., Williams K.W. (2013). Neuronal circuits that regulate feeding behavior and metabolism. Trends Neurosci..

[33] Sarvestani F.S., Tamadon A., Hematzadeh A., Jahanara M., Shirazi M.R., Moghadam A., Niazi A., Moghiminasr R. (2015). Expression of melanocortin-4 receptor and agouti-related peptide mRNAs in arcuate nucleus during long term malnutrition of female ovariectomized rats. Iran. J. Basic Med. Sci..

[34] Schwartz M.W. (2002). Neuronal pathways regulating food intake and body adiposity. Ann. Endocrinol. (Paris).

[35] Lanfray D., Richard D. (2017). Emerging Signaling Pathway in Arcuate Feeding-Related Neurons: Role of the Acbd7. Front. Neurosci..

[36] Smith M.A., Hisadome K., Al-Qassab H., Heffron H., Withers D.J., Ashford M.L. (2007). Melanocortins and agouti-related protein modulate the excitability of two arcuate nucleus neuron populations by alteration of resting potassium conductances. J. Physiol..

[37] Anderson E.J., Çakir I., Carrington S.J., Cone R.D., Ghamari-Langroudi M., Gillyard T., Gimenez L.E., Litt M.J. (2016). 60 YEARS OF POMC: Regulation of feeding and energy homeostasis by α-MSH. J. Mol. Endocrinol..

[38] Labbé S.M., Caron A., Lanfray D., Monge-Rofarello B., Bartness T.J., Richard D. (2015). Hypothalamic control of brown adipose tissue thermogenesis. Front. Syst. Neurosci..

[39] Contreras C., Gonzalez F., Fernø J., Diéguez C., Rahmouni K., Nogueiras R., López M. (2015). The brain and brown fat. Ann. Med..

[40] Butler A.A., Cone R.D. (2002). The melanocortin receptors: Lessons from knockout models. Neuropeptides.

[41] Won S.B., Jung G.Y., Kim J.H., Chung Y.S., Hong E.K., Kwon Y.H. (2013). Protective Effect of Pinus koraiensis Needle Water Extract Against Oxidative Stress in HepG2 Cells and Obese Mice. J. Med. Food.

[42] Boccellino M., D’Angelo S. (2020). Anti-Obesity Effects of Polyphenol Intake: Current Status and Future Possibilities. Int. J. Mol. Sci..

[43] Samodien E., Johnson R., Pheiffer C., Mabasa L., Erasmus M., Louw J., Chellan N. (2019). Diet-induced hypothalamic dysfunction and metabolic disease, and the therapeutic potential of polyphenols. Mol. Metab..

[44] Ibars M., Ardid-Ruiz A., Suárez M., Muguerza B., Bladé C., Aragonès G. (2017). Proanthocyanidins potentiate hypothalamic leptin / STAT3 signaling and Pomc gene expression in rats with diet-induced obesity. Int. J. Obes. (Lond.).

[45] Siegrist-Kaiser C.A., Pauli V., Juge-Aubry C.E., Boss O., Pernin A., Chin W.W., Cusin I., Rohner-Jeanrenaud F., Burger A.G., Zapf J. (1997). Direct effects of leptin on brown and white adipose tissue. J. Clin. Investig..

[46] Wagoner B., Hausman D.B., Harris R.B. (2006). Direct and indirect effects of leptin on preadipocyte proliferation and differentiation. Am. J. Physiol. Regul. Integr. Comp. Physiol..

[47] Atawia R.T., Bunch K.L., Toque H.A., Caldwell R.B., Caldwell R.W. (2019). Mechanisms of obesity-induced metabolic and vascular dysfunctions. Front. Biosci. (Landmark Ed.).

[48] Ahn S.M., Choe E.S. (2009). Activation of group I metabotropic glutamate receptors increases serine phosphorylation of GluR1 alpha-amino-3-hydroxy-5-methylisoxazole-4-propionic acid receptors in the rat dorsal striatum. J. Pharmacol. Exp. Ther..

[49] Kim S.M., Ahn S.M., Go B.S., Wang J.Q., Choe E.S. (2009). Alterations in AMPA receptor phosphorylation in the rat striatum following acute and repeated cocaine administration. Neuroscience.

